# Recent advances in poor HIV immune reconstitution: what will the future look like?

**DOI:** 10.3389/fmicb.2023.1236460

**Published:** 2023-08-07

**Authors:** Wenyuan Zhang, Lianguo Ruan

**Affiliations:** Department of Infectious Diseases, Wuhan Jinyintan Hospital, Tongji Medical College of Huazhong University of Science and Technology, Hubei Clinical Research Center for Infectious Diseases, Wuhan Research Center for Communicable Disease Diagnosis and Treatment, Chinese Academy of Medical Sciences, Joint Laboratory of Infectious Diseases and Health, Wuhan Institute of Virology and Wuhan Jinyintan Hospital, Chinese Academy of Sciences, Wuhan, Hubei, China

**Keywords:** HIV-1 infection, immunological non-responders, CD4^+^ T cells, immune reconstitution, therapeutic interventions

## Abstract

Combination antiretroviral therapy has demonstrated proved effectiveness in suppressing viral replication and significantly recovering CD4^+^ T cell count in HIV type-1 (HIV-1)-infected patients, contributing to a dramatic reduction in AIDS morbidity and mortality. However, the factors affecting immune reconstitution are extremely complex. Demographic factors, co-infection, baseline CD4 cell level, abnormal immune activation, and cytokine dysregulation may all affect immune reconstitution. According to report, 10–40% of HIV-1-infected patients fail to restore the normalization of CD4^+^ T cell count and function. They are referred to as immunological non-responders (INRs) who fail to achieve complete immune reconstitution and have a higher mortality rate and higher risk of developing other non-AIDS diseases compared with those who achieve complete immune reconstitution. Heretofore, the mechanisms underlying incomplete immune reconstitution in HIV remain elusive, and INRs are not effectively treated or mitigated. This review discusses the recent progress of mechanisms and factors responsible for incomplete immune reconstitution in AIDS and summarizes the corresponding therapeutic strategies according to different mechanisms to improve the individual therapy.

## 1. Introduction

The hallmark of infection with the human immunodeficiency virus (HIV) is the progressive destruction of CD4^+^ T cells in both count and function, particularly the activated CD4^+^ T cells. Loss of CD4^+^ T cells can cause opportunistic infections, non-AIDS-defining events (nADEs) and death in those who develop acquired immunodeficiency syndrome (AIDS; Deeks et al., 2013; Maartens et al., 2014). The development of combination antiretroviral therapy (cART) has achieved the suppression of viral replication and the increase in CD4^+^ T cell count in the majority of patients, leading to a significant decrease in morbidity of AIDS and mortality (Vieillard et al., 2016; Saag et al., 2020). Yet, 10–40% of people living with HIV (PLHIV) fail to achieve CD4 cell count reconstitution with cART, despite achieving suppression of HIV-1 viral load in the blood, and are referred to as immunological non-responders (INRs). Compared with immune responders (IRs) and sub-optimal immunologic responders (ISRs), INRs still have a higher incidence of AIDS-related fatalities and morbidity (Piconi et al., 2010; Lederman et al., 2011; Briceno et al., 2020; Shive et al., 2021; Wang L. et al., 2021; Zhao et al., 2021). In addition, chronic immune activation has been reported among PLHIV, elevating the risk of cardiovascular disease (Shah et al., 2018), metabolic syndrome (Dragović et al., 2021), kidney abnormalities (Swanepoel et al., 2018), and non-AIDS-related malignancies (Chammartin et al., 2021) compared with non-HIV infected individuals, causing significant negative effects on quality of life as well as mortality (Wilson and Sereti, 2013; Ipp et al., 2014; Sims et al., 2018). As an important global public health issue, incomplete immune reconstitution continues to adversely affect the survival quality of PLHIV. In this review, we highlight areas of recent advances in the mechanisms and risk factors of incomplete immune reconstitution and explore new targeted therapeutic interventions to improve immune restoration.

## 2. Complete and incomplete immune reconstitution

Immune reconstitution is broadly divided into two phases. First, after cART is initiated, HIV viral load declines, and lymphocytes are soon redistributed, leading to a rise in peripheral blood memory CD4^+^ T cells (CD45RO+). Naive CD4^+^ T cell (CD4^+^ CD45RA^+^CD62L^+^) regeneration enhances after treatment for 2–3 months which leads a slow and steady increase in CD4^+^ T cells without normal immune function. Second, after 3–6 months of late treatment, CD4^+^ T cells gradually recall their antigen-specific response to various antigens, such as cytomegalovirus (CMV) and tuberculin, except for HIV (Autran et al., 1997; Macdonald et al., 1998). Studies have reported that as viral load decreases, T lymphocyte activation markers like CXCL13, HLA-DR, and CD38 decline, promoting the immune system return to homeostasis (Autran et al., 1997; Li et al., 1998; Mehraj et al., 2019).

However, immune restoration is not always successful. Although viral load can be controlled at low or even undetectable levels with cART, there is considerable variability in terms of CD4^+^ T cell recovery. And this inconsistency between the decline in plasma viral load and the rise in CD4 count is influenced by multiple factors such as immune-related or pathogenic host-related which like abnormal immune activation and generally characteristics. People failing to completely restore CD4 T cell count are called immunological non-responders. To date, there is no consensus on the definition of “immunological non-responders.” Studies and guidelines generally define them in regard to confine CD4^+^ T cell count, increased CD4^+^ T cell counts from baseline, or confine percentage of CD4^+^ T cell increase over baseline (Yang et al., 2020; Table 1). In 2008, the Department of Health and Human Services (DHHS) defined “immunological non-responders” as patients whose CD4^+^ T cell counts had not reached 350–500 cells/μl after 4–7 years of effective antiretroviral therapy. In 2021, the World Health Organization (WHO) defined adult immunological failure as a CD4 cell count of 250 cells/μl or a CD4 cell count consistently below 100 cells/μl after 6 months of effective treatment (WHO, 2021). In 2021, DHHS defined “immunological responders” as patients with an increase in CD4 count of 50–150 cells/μl in the first year of cART treatment. In the first 3 months, the response of treatment was rapid, followed by an average annual increase of approximately 50–100/μl until steady state (Adolescents., P.O.A.G.F.A.A, 2021).

**Table 1:** Definitions of INR from the literature and guideline.

Definition of INRs	References
Increase in the CD4^+^ T cell count <200 cells/μl from baseline at 2 years after cART initiation, with an undetectable plasma VL	García et al. (2019)
Total CD4^+^ T cell counts <200 cells/μl at 2 years after cART initiation, with an undetectable plasma VL	Xie et al. (2021)
Total CD4^+^ T cell count <350 cells/μl 2 years after cART initiation, with an undetectable plasma VL	Dai et al. (2021), Rousseau et al. (2021), Shive et al. (2021)
Total CD4^+^ T cell count <350 cells/μl 2 years after cART initiation, with plasma HIV-1 RNA < 50 copies/ml	Geng et al. (2021), Malazogu et al. (2021), Vlasova et al. (2021)
Total CD4^+^ T cell count <350 cells/μl 2 years after cART initiation, with plasma HIV-1 RNA < 50 copies/ml at least 12 months	Bandera et al. (2018)
Total CD4^+^ T cell counts <350 cells/μl 8 years after cART initiation, with an undetectable plasma VL	Luo et al. (2022)
Increase in the CD4^+^ T cell count <20% from baseline at 1 year after cART initiation, with an undetectable plasma VL	Lv et al. (2021)
CD4/CD8 ratio < 1 at 24 weeks after cART initiation, with plasma HIV-1 RNA < 50 copies/ml	Russo et al. (2022)

VL, viral load.

INRs are unable to maintain the basic normal immune function of the body due to low CD4^+^ T cell counts and exhibit severe immune dysfunction. Under the influence of lifestyle, drug toxicities, chronic inflammation, immune activation, and many other factors, nADEs still account for an increasing proportion of PLHIV, despite viral load being controlled to undetectable levels after cART (Elvstam et al., 2021). However, the normal CD4 counts could not always reflect complete restore, though it be extensively applied in evaluating immune reconstitution (Ron et al., 2023). Recently, CD4/CD8 ratio was demonstrated having an amazing performance in immune reconstitution. Some studies insisted that a low CD4/CD8 ratio reflects increased immune activation and immune senescence, also associating with an higher risk of severe nADEs (Gibellini et al., 2017; Serrano-Villar et al., 2022). Therefore, we suggested incomplete immune reconstitution an independent risk factor for increased risk of SNAEs under sustained viral suppression (Prabhu et al., 2019; Noiman et al., 2022).

## 3. Potential mechanisms of incomplete immune reconstitution

**Figure 1:**
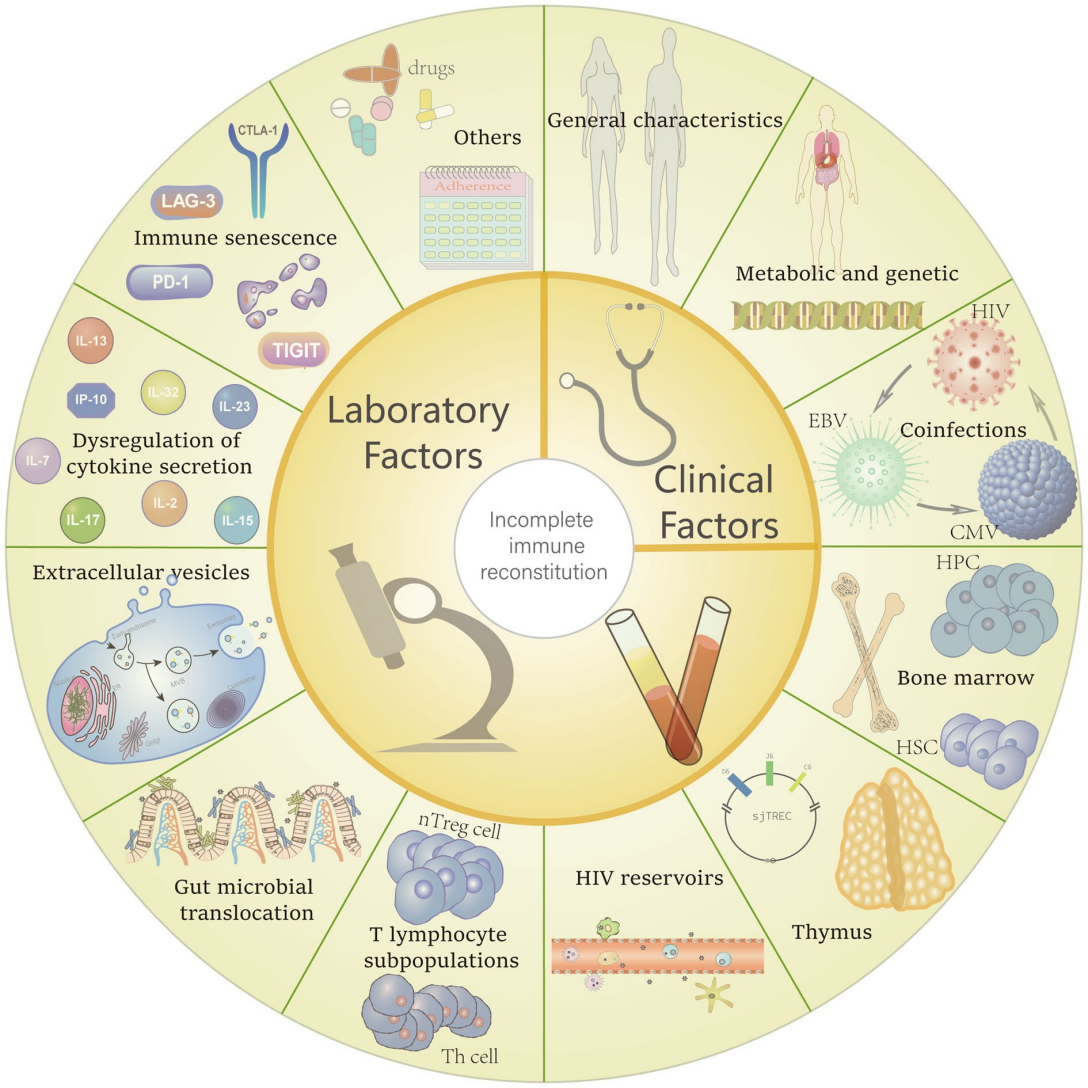
Potential risk factors for incomplete immune reconstitution. Including general characteristics (e.g., age, gender, CD4^+^ T-cell counts, body mass index (BMI) and the underlying diseases), metabolic and genetic factors also be confirmed can make some difference in immune recovery between different people living with HIV (PLHIV). Furthermore, coinfections with other virus, poor adherence and the regimens which with severe side effects also can interfere the immune restore. Factors that contribute to the reduction and destruction of CD4^+^ T cells also affect immune reconstitution, including: reduced haematopoiesis of bone marrow, thymic insufficiency, replication of HIV reservoirs, alteration of CD4^+^ T cell subpopulations, gut microbial translocation, transport effects of extracellular vesicles, dysregulation of cytokine and, immune senescence.

Failure to normalize the reduced CD4^+^ T cell population after treatment is regard as incomplete immune reconstitution. This outcome results from decreased production, increased destruction, and increased senescence of CD4 cells. The mechanisms that cause these abnormal changes in CD4 cells are currently the subject of much speculation. Such as damage and hypofunction of lymphoid organs, residual viral replication, disruption of T cell subpopulation homeostasis, dysregulation of cytokine secretion, and translocation of microorganisms might reduce CD4^+^ T cell count directly and induce inflammation and immune activation. It is worth to mention that excessive activation of CD4^+^ T cells during cART has been suggested to upregulate the expression of the HIV-1-binding target CCR5 on CD4^+^ T cells, thereby accelerating the depletion of CD4^+^ T cells (Gandhi et al., 2017; Xia et al., 2018). Therefore, long-term chronic abnormal immune activation can also affect immune reconstitution. In addition, factors associated with the host can also affect CD4 cell recovery (Figure 1).

**Figure 2:**
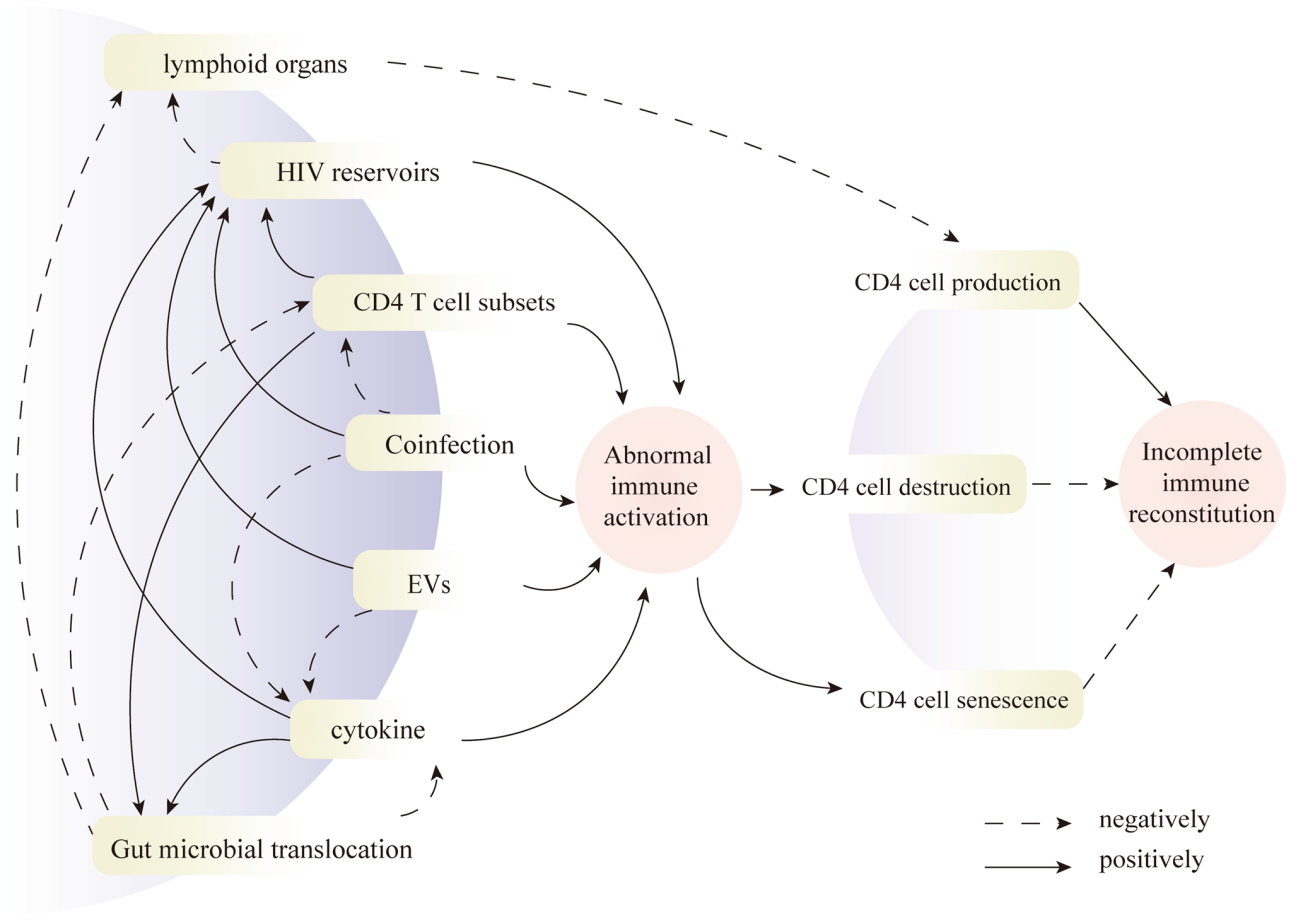
Pathways contribute to poor immune reconstitution from the above factors.

To this end, we discuss herein the possibilities affecting incomplete immune reconstitution and its mechanisms (Figure 2).

### 3.1. Dysfunction of the lymphoid organs

T cells are produced from hematopoietic progenitor cells (HPCs) and hematopoietic stem cells (HSCs) in the bone marrow, educated on thymic tissue for development and maturation. Hematopoietic stem and progenitor cells (HSPCs) have the capacity for lifelong survival, self-renewal, and daughter cell production. HIV-1 infection can lead to impaired hematopoietic function of HSPCs in several ways. First, a large body of evidence indicates that low levels of CD4 receptors expressed by HSPCs can be targeted by HIV thereby leading to impaired function (Sebastian et al., 2017; Chen, 2019; Karuppusamy et al., 2022). Second, HIV-1-produced Nef proteins expressed in HSPCs impeding the development and production of myeloid–erythroid cells and inducing apoptosis (Zou et al., 2021). In addition, HIV-1 is capable of causing alterations to the bone marrow hematopoietic microenvironment. HIV-1 may complete with stromal cell-derived factor-1 (SDF-1, also known as CXCL12) for the site of binding to CXCR4, affecting the homing of HSCs (Tsukamoto, 2020). However, efficient homing is a prerequisite for the successful re-establishment of hematopoiesis in the bone marrow hematopoietic microenvironment. Furthermore, Yuan et al. (2019) found that HIV-Tat reduced the ability of bone marrow mesenchymal stem cells (BMSCs) to support HSC expansion *in vitro* and reduced the expression levels of a series of key hematopoietic factors produced by BMSCs. These studies suggest that incomplete immune reconstitution in HIV-1-infected patients may be associated with reduced bone marrow hematopoietic function and HSPCs numbers.

The thymus is the site of T cell development and maturation, and good thymus function ensures the stability of CD4^+^ T cell count and function. As a marker of thymic function, CD31^+^CD4^+^ T cells (CD31%) reflects the kinetics of the recent thymic immigrant CD4^+^ T cells. In a research, the results revealed a lesser percentage of CD31% in the INR group than in the IR group, and both of them exhibited significantly lesser percentages than the healthy volunteer group (Li et al., 2011). Ferrando-Martinez et al. (2017) applied sj/β-TREC (accurate technique for measuring thymic function) to 774 PLHIV of different types and found that patients with thymic failure (sj/β-TREC ratio < 10) had significantly lower levels of CD4^+^ T cells, suggesting that thymic function influences AIDS progression. Recent thymus emigrants (RTE) is one of the most established markers to evaluate recent thymic output function, representing a high sj-TRECs content CD4^+^ T cell subpopulation (Kimmig et al., 2002). Briceno et al. (2020) identified that HIV-positive subjects had fewer RTEs in absolute terms compared with the HIV-negative group. The application of cART resulted in increased levels of circulating RTEs in IRs compared with those in INRs. A further study (Rb-Silva et al., 2019) reported that IRs demonstrated a significant increase in thymus volume at 12 months of cART treatment, were prone to higher sj-TREC and sj/β-TREC, and had higher proportions and absolute numbers of RTEs in peripheral blood CD4^+^ T cells than INRs. These studies suggest that the thymus influences HIV progression by affecting CD4^+^ T cell levels, and impaired thymic output and reduced function are one of important mechanisms leading to incomplete reconstitution.

### 3.2. Replication of residual virus in HIV reservoirs

HIV-1 invades the body and preferentially attacks activated CD4^+^ T cells, most of which die rapidly after infection, whereas a small proportion, called latent cells, tend to enter a resting state and stay dormant for a long time. HIV-1 proviruses have the capacity for stable integration and high replication and can persist *in vivo* in cells (Wacleche et al., 2018; Zaikos et al., 2018; Kruize and Kootstra, 2019; Wallet et al., 2019; Lutgen et al., 2020; Wiche Salinas et al., 2021; Chen et al., 2022b) and tissues (Damouche et al., 2015; Cantero-Pérez et al., 2019; Ganor et al., 2019; Wallet et al., 2019; Chaillon et al., 2020a) of PLHIV receiving cART. Reactivation of these latent proviruses is regarded as the leading cause of rebound viremia after cART cessation (Vanhamel et al., 2019; Cohn et al., 2020). We found a majority of people on suppressive cART have residual virus production, which may cause immunological activation as a result of the anti-HIV immune response. Therefore, the increased immune activation and inflammation in those using cART appears to be mostly caused by HIV persistence (Scherpenisse et al., 2021). Mavigner et al. (2009) investigated the connection between residual virus and T-cell activation in both groups of good immunological responders and incomplete immunological responders. In individuals with incomplete immunological responses, there was a strong correlation only between the degree of residual viremia and the frequency of CD4 T-cell activation. Furthermore, they also found the prevalence in CD31+ naive CD4 T cells of RTEs seemed to be inversely linked with the residual viremia (Mavigner et al., 2009). The findings of these studies imply that the presence of prolonged HIV-1 replication *in vivo* provides a basis for viral rebound while leading to a long-term chronic reduction in CD4 T cells.

### 3.3. Breakdown of CD4 T cell subsets

During chronic HIV infection, a slow and sustained decline in circulating CD4 T cells is accompanied by changes in major CD4 cell subsets. Regulatory T cells (Treg) are one of the most important elements in suppressing immune responses, which can both control aberrant immune activation and suppress specific T cell responses to a variety of pathogens for causing immunodeficiency, critical in maintaining immune homeostasis (Chen Y. et al., 2020). As they express the CCR5 receptor, Tregs are vulnerable to HIV infection, and the interaction of gp120 and CD4 inhibits Treg apoptosis and improves its survival. Therefore, Treg is also a potential reservoir for the HIV-1 virus (Nilsson et al., 2006; Sivanandham et al., 2020). In addition, Tregs are capable of controlling low residual immune activation in virologically suppressed patients under cART. A previous study (Chevalier and Weiss, 2013) found that the proportion of Tregs under cART treatment was negatively correlated with CD 4 and CD8 T cell activation. Thus, under viral control, Treg frequency is negatively correlated with residual immune activation. While the early initiation of cART restored CD4^+^ T cell count, it was unable to control the massive expansion and intestinal migration of Treg, which may eventually cause HIV disease progression and intestinal tissue fibrosis (Yero et al., 2019, 2021).

Helper T cells (Th) are the most numerous subsets of T cells and work by amplifying the immune response function of other immune cells. HIV preferentially infects Th1 and Th17 cells. Among them, Th17 cells are enriched within the mucosal tissue and promote protective immune responses against extracellular bacteria and fungi, preserving the integrity of the mucosal barrier and maintaining immune homeostasis at mucosal sites (Planas et al., 2019; Wiche Salinas et al., 2021). PLHIV commonly exhibits a progressive decline in intestinal CD4^+^ T cells, with a preferential absence and significant depletion of Th17 cells, throughout infection (Le Hingrat et al., 2021). Depletion of Th17 cells has important implications for disease progression and viral persistence and is a major cause of microbial translocation and inflammatory progression (Ortiz et al., 2016). At the same time, Th17 cells are closely associated with Treg and altered Th17/Treg balance is associated with a persistent chronic inflammatory state in HIV-1 disease (Chevalier and Weiss, 2013). Chronic inflammation has long been known to be harmful in conditions when the immune system is unable to successfully remove infectious germs. Therefore, chronic HIV-1 infection activated the host immune system and attacked the virus through multiple mediators. Then, it led to deprivation of T-cell function and ultimately to immunosuppression (Favre et al., 2010). The alterations in T cell subsets can affect immune activation through multiple pathways leading to incomplete control of viral replication and facilitating the progression of immune activation. And now, how to detect changes in each subpopulation and discover their role is crucial to our next step of targeted preventive and control for this progression.

### 3.4. Gut microbial translocation

The gut-associated lymphoid tissue contains at least 80% of the body’s lymphocytes, including approximately 60% of the body’s CD4^+^ T lymphocytes, making the intestinal mucosal immune system a major target of HIV-1 attack, and the body’s immune function is closely related to the intestinal microenvironment (Brenchley and Douek, 2008). Microbial translocation can lead to changes in species diversity and composition, affecting systemic inflammation and thus CD4 T cell recovery.

In healthy individuals, a wide variety of microorganisms reside in the gut, including beneficial and pathogenic bacteria, which together participate in various host physiological functions and maintain normal physiological activities. Lee et al. (2018) found that *Lactobacillus* is involved in maintaining the integrity of the intestinal mucosal barrier. It reduces microbial translocation, has a role in reducing systemic immune activation, and its abundance is positively correlated with CD4^+^ T cell count. Another study confirmed that *Bacteroides* has LPS or LOS molecules capable of signaling through TLR4 to induce IFN-β to initiate antiviral responses and resistance to viral infection (Stefan et al., 2020).

However, HIV-1 infection leads to a disruption in the bacterial community structure and function of the intestinal microbiota, primarily characterized by an overall decreased α diversity, a significant increase in relative abundance of *Prevotella* and reduced abundances of *Bacteroides* and *Faecalibacterium* (Vujkovic-Cvijin et al., 2013; Dillon et al., 2014; Mak et al., 2021). These changes in the gut microbiota can stimulate the activation of T cells and chronic inflammation directly or indirectly, leading to systemic immune activation (Vujkovic-Cvijin and Somsouk, 2019; Geng et al., 2020; Serrano-Villar et al., 2021). Ishizaka et al. (2021) observed that compared with healthy controls, PLHIV with low CD4 counts had reduced gut microbiome α diversity and a higher abundance of *Prevotella*, and the relative abundance of it was positively correlated with inflammatory cytokines and negatively correlated with anti-inflammatory cytokines. A recent study concluded that *Prevotella* increased the susceptibility to gut inflammation through the suppression of IL-18 production indirectly (Iljazovic et al., 2021). The findings of Pinacchio et al. (2020) suggest that *Prevotella* levels were negatively correlated with IFN-I gene expression and Th17 cell counts. It is further hypothesized that *Prevotella* enrichment may affect intestinal mucosal IFN-I pathways and T cell response in HIV-1-infected patients, therefore leading to immune dysfunction. Another study (Pickard et al., 2017) suggested that gut microbes can induce Th17 and Treg production by modulating immune cell differentiation. In addition, some studies have demonstrated that gut microbes can regulate myeloid hematopoietic cell development and maturation by modulating local metabolites and tissue-specific mediators as well as by driving Toll-like receptor (TLR) and myeloid differentiation factor 88 (MyD88)-mediated signaling pathways (Khosravi et al., 2014; Serrano-Villar et al., 2016; Thaiss et al., 2016). The results of these studies suggest that gut microbes have a role in the regulation of T-cell subsets and bone marrow hematopoiesis. The above findings suggest that alterations in the gut microbiota may be associated with abnormal immune activation and chronic inflammation during chronic HIV-1 infection. We suggest that after HIV-1 invasion, intestinal mucosal lymphocytes, the primary target of attack, are depleted by direct viral action and post-activation apoptosis, and the intestinal mucosal barrier is compromised, indirectly leading to dysbiosis/microenvironmental changes in the intestinal flora. These changes in turn further exacerbate the damage to the intestinal mucosa, leading to an increased risk of inflammation and promoting abnormal immune activation.

### 3.5. HIV-1 infected extracellular vesicles changed structure and function, interfering immune reconstitution

Extracellular vesicles (EVs) are heterogeneous phospholipid bilayer vesicles released from cells into the external environment and act as specific carriers and mediate intercellular communication in the immune system (Théry et al., 2018; Gudbergsson et al., 2019). Furthermore, they are involved in the regulation of immune responses in different physiologic and pathogenic settings, by delivering nucleic acids, lipids, and proteins from their surface or lumen to target cells (Cheruiyot et al., 2018; Witwer and Théry, 2019).

First, EVs can contribute to HIV transmission and HIV reservoirs in direct and indirect ways. Several studies have demonstrated that large numbers of EVs released from HIV-1-infected cells are involved in HIV-1 transmission under their carrier properties, and cART is unable to reduce their concentration levels (Lee et al., 2016; Patters and Kumar, 2018). Weber et al. (2020) treated cultured monocytes with HIV-Tat *in vitro* and found that monocyte-derived microvesicles (MDMVs) released by Tat-treated monocytes into the culture medium were significantly increased. In addition, another HIV protein, Nef, can promote HIV infection by decreasing the expression of CD4 in exosomes to improve the ability for neutralize with CD4-bearing cells (Navarrete-Muñoz et al., 2021). Another study (Perez et al., 2019) found that EVs secreted by infected cells carry pathogen-associated molecular patterns, which that interact with immune cells to produce inflammation and also evade recognition by the immune system. EVs can also expand the number of susceptible target cells by mediating the transfer of CXCR4 and CCR5 to target cells that do not express or low express these molecules.

Secondly, EV can promote immune cell activation and secondary inflammatory responses leading to failure of immune reconstitution. Okoye et al. (2021) noted that plasma extracellular vesicles from HIV-1 patients could induce miR-139-5p expression to promote CD4^+^ T cell activation. Gabriel et al.(Duette et al., 2018) observed that T cells in the HIV-1 replication cycle can enhance viral replication by inducing hypoxia-inducible factor 1α (HIF-1α) activity. They also noted that HIF-1α induced the release of EVs, causing associated inflammation to stimulate the secretion of inflammatory mediators IL-6 and IL-1β by uninfected lymphocytes and macrophages, further promoting viral replication and inflammation. EVs are involved in HIV-1 and intercellular communication, contributing to the spread of the virus between cells. The presence of this modality greatly increases HIV-1 latency and also results in the inability of cART to completely clear the virus, increasing the risk of inducing an inflammatory response.

### 3.6. Dysregulation of cytokine secretion

Interleukin-2 (IL-2) promotes the activation and proliferation of T lymphocytes and NK cells, stimulates the secretion of various cytokines, and provides important common signals for the specific immune response. As a result of HIV-1 infection, the production of IL-2-expressing memory CD4^+^ T cells is reduced, leading to decreased IL-2 levels. This is one of the earliest functional defects observed in chronic PLHIV and predicts the loss of CD4^+^ T cells and the progression of AIDS (Xia et al., 2018).

IL-7 and IL-15 are the primary stimulators for the production of naive and memory T cells during chronic infection, maintaining T cell function and internal environmental stability (Nasi and Chiodi, 2018; Harwood and O'Connor, 2021; Simonetti et al., 2021). However, the persistent low proliferative response of CD8^+^ T cells and Th17 CD4^+^ T cells to IL-7 after cART affects immune dysfunction among PLHIV (Côté et al., 2020). However, by observing IRs and ISRs after cART, Pino et al. (2021) found that ISRs had higher plasma levels of IL-7 and IL-15 before cART compared with IRs, which in turn supported homeostatic proliferation of T cells. This paradox led us to speculate that elevated levels of IL-7 and IL-15 in ISRs might also promote the persistence of HIV-1 viral hosts by enhancing cell survival. This has the same results as chemokine IP-10, which exerts a pro-inflammatory effect by triggering the activation of cofilin and actin to enhance the kinetic activity of quiescent CD4^+^ T cells and induce viral entry, thereby promoting latent HIV infection (Wang Z. et al., 2021).

IL-17 is mainly produced by Th17 and Tc17 cells, and can encourage the integrity of the intestinal mucosal barrier by promoting tight junctions of intestinal epithelial cells, secreting antimicrobial peptides, and summoning neutrophils to the sites of mucosal injury (Liang et al., 2006; Lee et al., 2015). During HIV-1 infection, the number and function of IL-17-producing Th17 cells and Tc17 cells are impaired in the peripheral and intestinal mucosa due to direct viral infection and impaired IL-23 signaling pathways that maintain Th17 cell production and stability, and IL-17 levels are reduced, exacerbating impaired intestinal barrier integrity and thus causing microbial translocation and immune activation (Fernandes et al., 2017; Perdomo-Celis et al., 2018). Furthermore, Moreira Gabriel et al. (2021) found that overexpression of IL-32b mRNA in the colon of HIV-1 patients receiving cART had a negative correlation with IL-17A mRNA levels, suggesting that overexpression of IL-32 may also affect the integrity of the intestinal epithelial barrier.

Studies have reported that PLHIV has higher levels of pro-inflammatory cytokines such as IL-1β, IL-6, IL-8, and IL-18 compared with healthy individuals, which are associated with reduced CD4+ T cell recovery at month 12 after cART treatment (Vanpouille et al., 2020; Nganou-Makamdop et al., 2021). In addition, Wang et al. (2020) found that the persistent inflammatory response caused by HIV-1-induced cytokine production leads to a reduction in the intestinal mucosa in ILCs. ILCs do not express markers or receptors such as CD4 or CCR5, even with the application of cART, ILCs in blood and intestinal were depleted. That also led to a loss of intestinal epithelial integrity and microbial translocation, further exacerbating chronic inflammation.

Cytokines regulate cell growth and differentiation, immune response, and inflammatory response. Excessive immune activation leads to persistent chronic inflammation, causing loss of T-cell function and ultimately immunosuppression.

### 3.7. Premature immune senescence due to increased T cell apoptosis and senescence

T-cell-mediated cellular immunity is the primary mode of defense against foreign pathogens. However, with prolonged and sustained exposure to chronic stimuli such as chronic infections and cancer, T cells can enter a state of dysfunctional failure if the immune response is unable to fully clear foreign antigens. This failure is first manifested in PLHIV by a progressive decline in the killing function of CD8^+^ T cells (Barber et al., 2006; Okoye and Picker, 2013). Without CD8^+^ T cell suppression, viral load rises rapidly, and as antigen levels continue to rise, T cells, which are the target cells of HIV-1, are depleted and gradually failed to kill infected cells effectively (Collins et al., 2021). Meantime, the body’s negative regulatory factors are highly expressed in CD4^+^ and CD8^+^ memory T cells, including a variety of inhibitory receptors associated with T cell failure, also known as immune checkpoint inhibitors (ICIs), such as programmed cell death 1 (PD-1), lymphocyte-activation gene 3 (LAG-3), cytotoxic T lymphocyte antigen 4 (CTLA-4), and the newly identified T-cell immunoglobulin and ITIM domains (TIGIT) and T cell immunoglobulin and mucin-domain containing protein 3 (TIM3; Minnie et al., 2018; Dixon et al., 2021; Kubli et al., 2021; Tawbi et al., 2022). These ICIs negatively regulate T cell function by binding to their respective ligands to generate-T cell-suppressive signals, which associate with viral load and CD4^+^ and CD8^+^ T cell counts and dysfunction, reflecting to some extent the state of T cell failure (Fenwick et al., 2019).

### 3.8. Coinfection

In cART, HIV is often combined with other types of viral infections due to low immunity, and studies have found that hepatitis B virus (HBV), hepatitis C virus (HCV), cytomegalovirus (CMV), Epstein–Barr virus (EBV), and other viruses may affect CD4+ T cell recovery and immune reconstitution. Plasma sCD163 and sCD14 are sensitive indicators of the level of response to chronic immune activation and have a significant relation with the progression of HIV and HBV (Wilson et al., 2014; Shive et al., 2015). He et al. (2021) found both sCD163 and sCD14 presented elevated expressions in groups with coinfected and single-infected patients compared with those in the healthy group. However, compared with HIV-1 single-infected patients, sCD14 expression remained at a high level after reduction in HIV-1/HBV-coinfected patients, and the Treg cell frequency was significantly higher than the former, at week 24 of cART. It has been reported that PLHIV (Garcia-Broncano et al., 2018; Gobran et al., 2021) were noted to be six folds more likely to be exposed to HCV and get infected compared normal people. The infection affects the stability of CD4^+^ T cell count negatively while promoting HIV-1 replication and viral reservoir persistence. Moreover, several studies have affirmed that HIV-1/HCV-coinfected individuals presented a remarkable reduction in the median expression of HLA-DR and CD38 on CD4 and CD8 T cells, as HCV is controlled. Meanwhile, after HCV eradication, significantly lower levels of innate immune activation markers (sCD163, sCD14), HIV-1 proviral load and LPS (López-Cortés et al., 2018; French et al., 2021). Furthermore, the coexistence of CMV or EBV with HIV-1 in PLHIV also contributes to elevated plasma inflammatory factors and promotes chronic immune activation, which is a key element for T cell activation and incomplete immune reconstitution after cART (Hernandez et al., 2018). Chaillon et al. (2020b) observed a significant increase in the diversity of HIV-1 DNA molecules among partitions with high CMV and EBV transmission, thus suggesting that sustained CMV and EBV replication and associated inflammation may maintain the persistence of HIV-1 hosts. These studies suggest that HIV-1 in combination with other viral infections leads to disruption of T cell subpopulation homeostasis, increased HIV-1 replication, and abnormal activation of CD4^+^ T and CD8^+^ T cells, further causing massive CD4^+^ T cell destruction and a chronic inflammatory response.

### 3.9. Host-associated factors

In addition, some of the risk factors for immune reconstitution recovery include older age (Kroeze et al., 2018; Ahn et al., 2019; Chen et al., 2022a), males (Boatman et al., 1999; Fiseha et al., 2022), low CD4^+^ T cell baseline level (Davy-Mendez et al., 2019; Handoko et al., 2020)，delay in cART initiation (Sharma et al., 2019; Saluzzo et al., 2021; Jesson et al., 2022)，inappropriate cART regimen (Veil et al., 2020; Lu et al., 2022), adherence, and the underlying diseases.

In addition, host metabolic level and genetic factors play a role in immune reconstitution. Studies (Zhu et al., 2022) have demonstrated that PLHIV had better immune reconstitution with a higher baseline BMI. This may be due to the fact that leptin has higher levels in people with a higher BMI. As an adipokine regulatory protein, leptin play an essential role in acquired immunity (de Candia et al., 2021). Many researches pointed that abnormalities in leptin synthesis contribute to the dysregulation of T-cell immunity (Batra et al., 2010). Therefore, we speculate that a lower BMI may predicts an incomplete immune reconstitution. Furthermore, hereditary variables of the organism have been suggested to influence immune reconstitution. A previous study (Pereira et al., 2022) HLA class 1 B*13, B*35 and B*39 alleles were linked to influence recovery failure. And even while cART is being used, HLA can facilitate the loss of CD4 T cells in non-responders due to the uncontrolled immunological activation in HIV infection.

## 4. Interventions for poor HIV immune reconstitution

**Figure 3:**
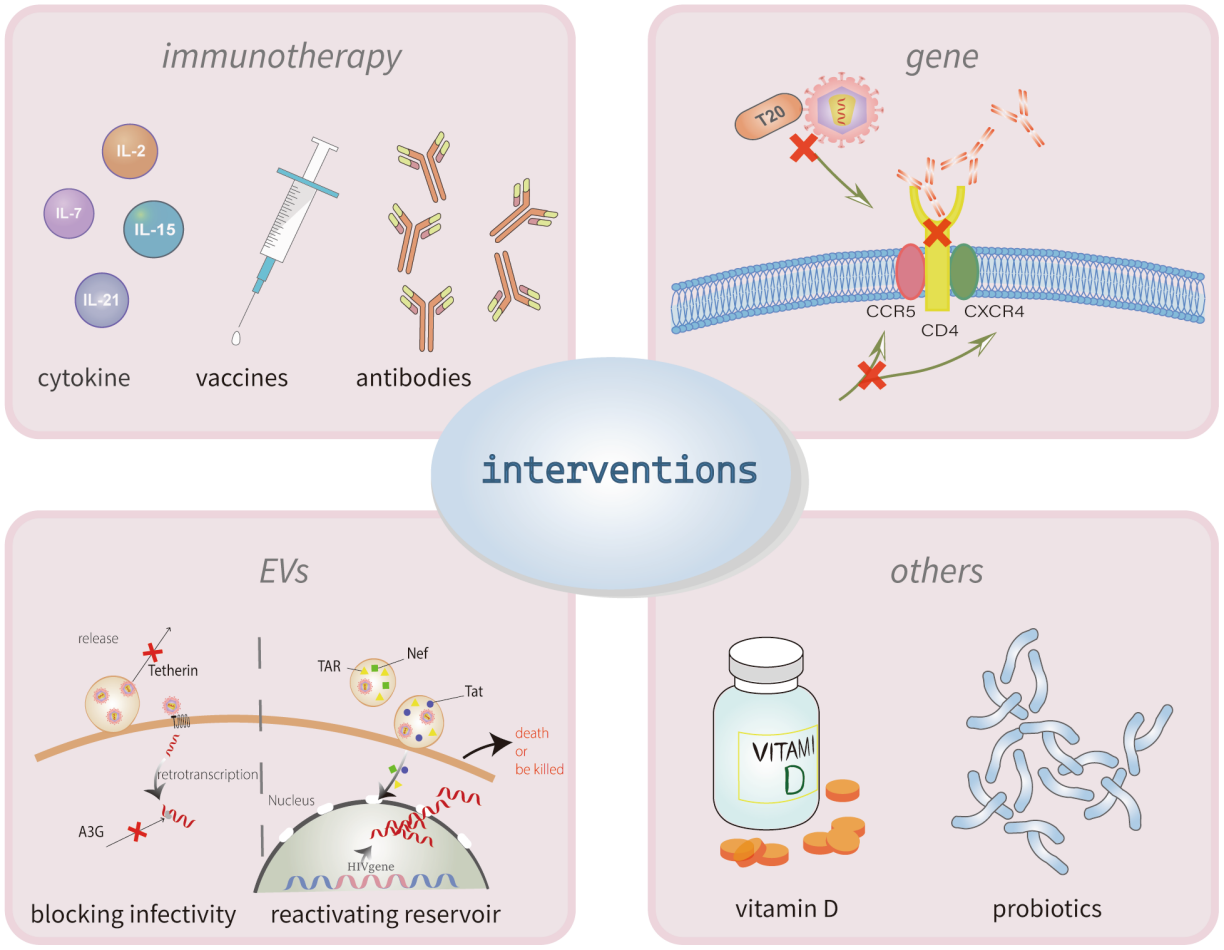
Interventions for poor HIV immune reconstitution. The mechanisms of incomplete immune reconstitution in PLHIV have not yet been clarified. The current intervention strategies for recover the CD4^+^ T cells mainly through two methods, immunotherapy and gene therapy. Immunotherapy can use the cytokines, vaccines or antibodies to activate latent cells and enhancing immune killing capacity. Similarly, treatments of targeting the anti-HIV-1 gene could make target cells resistant to viral infection. Other treatments to modulate immune also have some effect, like the application of vitamin D and probiotics.

Since the residual virus cannot be completely removed from the body, once cART is started it is a lifelong treatment. However, some patients do not recover well after treatment, and at the same time, long-term adherence problems and adverse effects lead to poor outcomes in an increasing number of patients, which affects the progression of the disease. Heretofore, there is no definitive treatment for patients with incomplete immune reconstitution, but there are many attempts and trials to improve immune reconstitution and slow down disease progression (Figure 3).

### 4.1. Immunotherapy

The prime goal of immunotherapy, a novel alternative strategy, is to induce immune recovery, reduce pathogenicity, reduce HIV-1 inflammation and immune activation, promote a direct and effective specific immune response, and normalize the immune system. The main approaches currently available include cytokine therapy, therapeutic vaccines, and targeted antibody therapy.

Some cytokines appear to improve CD4^+^ T cell count in modulating immunity. Many studies have found that IL-7 does not cause significant changes in plasma viral load while amplifying CD4^+^ and CD8^+^ T cells (Vassena et al., 2012; Thiébaut et al., 2016; Steele et al., 2017). Thus, the application of recombinant human interleukin-7 stimulates the proliferation of CD4^+^ T cells in circulating blood and helps INRs to re-establish immune function (Logerot et al., 2018; Wang et al., 2018). Moreover, Goshu et al. (2020) and Chen P. et al. (2020) found that rhIL-15/PD-L1 antibody combination therapy resulted in a transient increase in Ki67^+^ NK cells and CD8+ T cells as well as enhanced CD8^+^ T cell function safely and endurably, with no significant increase in plasma viral load after cART interruption (Okoye et al., 2019). In rhesus monkeys infected with the Simian immunodeficiency virus, Harper et al. (2021) found that combination therapy with IL-21 and IFNα during cART was effective in promoting NK cell terminal differentiation maturation and production of IFN-γ, which contributed to the clearance of virus from tissue cells.

Presently, HIV-1 vaccines can be categorized into therapeutic and preventive vaccines. In 2009, a DNA vaccine, ALVAC-HIV+, was reported to prevent HIV-1 infection by eliciting antibody-dependent cytotoxic effects, and numerous clinical trials have shown efficacy rates of up to 31.1% (Rerks-Ngarm et al., 2009). However, data from a recent phase III clinical replication trial (HVTN702) showed no protective effect in the vaccination group compared with the control group (Gray et al., 2021). Furthermore, the main peptide vaccines currently available are C4-V3 polyvalent peptide vaccine (Vieillard et al., 2016), Vacc-4x (Pollard et al., 2014), and VAC-3S (Vieillard et al., 2019), and there are also several prophylactic or therapeutic dendritic cell vaccines, such as Dec205 (Hossain and Wall, 2019; Matsuo et al., 2021) and Clec9A (Canton et al., 2021), Ags-004 (Gay et al., 2018), and DCV-2 (Espinar-Buitrago and Muñoz-Fernández, 2021).

Tat proteins can influence the activation and latency of the HIV-1 reservoir, thereby determining disease progression. A few studies have reported that anti-Tat immunization during infection or cART may inhibit potential HIV reactivation, thereby reducing persistent low-level viremia and T cell activation and promoting CD4^+^ T cell recovery, with positive effects on the long-term kinetics of immune reconstitution in individuals on long-term cART therapy (Sgadari et al., 2019; Tripiciano et al., 2021). A study found that HIV-1 gp120 envelope V1V2 region epitope-specific V2p and V2i antibodies failed to neutralize HIV-1 in plasma effectively but could mediate Fc segment-dependent antiviral activity, thereby inducing a more focused functional V2p and V2i-specific antibody response such as antibody-dependent cell-mediated cytotoxicity and antibody-dependent cell-mediated phagocytosis (Weiss et al., 2022). It has been reported frequently that anti-HIV-1 broadly neutralizing antibodies (bNAbs), such as 3BNC117 (Gaebler et al., 2022), 10-1074 (Gaebler et al., 2022), and VRC01 (Bar et al., 2016; Corey et al., 2021), are safe and effective for maintaining viral suppression in a short period and can be used as an adjunctive therapy to cART.

The main immunotherapeutic strategies currently available are targeting the inducible activation of latently infected cells and enhancing their own immune killing capacity. Although numerous researchers have been fruitful in this field over the years, whether the desired effect on HIV clearance mechanisms can be achieved remains unanswered.

### 4.2. Gene therapy

With two HIV-infected patients successfully achieving a “functional cure” after receiving CCR5Δ32D allele-pure HSC transplants (Hütter et al., 2009; Gupta et al., 2019; Claireaux et al., 2022), scientists believe that HIV-1 target cells can be made resistant to viral infection by targeting the anti-HIV-1 gene. The three main types of gene therapy currently under investigation are blocking co-receptor binding, blocking CD4 binding, and blocking membrane fusion.

Co-receptors play a critical role in the entry of HIV-1 into CD4^+^ cells. Scientists have conducted numerous trials and found that T cells infused with zinc finger nucleases disrupted the CCR5 gene giving transgenic cells a survival advantage and a good safety profile (Tebas et al., 2014, 2021).

Ibalizumab, a humanized IgG4 monoclonal antibody, blocks HIV-1 binding to chemokine receptors to inhibit viral entry by binding non-competitively to the CD4 receptor and causing deformation of the CD4-gp120 complex (Jacobson et al., 2009; Beccari et al., 2019; Kufel, 2020). Several clinical trials (Gathe et al., 1999; Emu et al., 2018) have shown that significant antiviral activity of Ibalizumab in advanced disease and multidrug-resistant PLHIV with limited treatment options.

Enfuvirtide (T20) is a synthetic peptide antiretroviral drug and the only entry inhibitor approved by the Food and Drug Administration (FDA) for the treatment of HIV-1. As a mimic of the HIV-1 glycoprotein gp41, enfuvirtide binds to the viral envelope and interferes with membrane fusion by interfering with the conformation required for viral invasion into the host cell, blocking viral entry into the host cell (Carr, 2003; Lalezari et al., 2003).

Although the application of gene therapy in HIV treatment still faces some limitations, more strategies need to be developed in order to improve the therapeutic power. Effective gene modification options, smart targeting combined with CAR T-cell therapy, and novel genome editing strategies, are novel future research directions (Sheykhhasan et al., 2021).

### 4.3. Extracellular vesicles therapy

At present, EVs control HIV-1 infection mainly via two aspects: blocking HIV infection and reactivating the HIV reservoir. Tetherin proteins that mediate MVs transport and contact with receptor membranes can keep microvesicles arrested on the cell surface prevent the release and transmission of HIV-1-carrying extracellular vesicles and, to a certain extent, alleviate the associated inflammatory disease (Weber et al., 2020). A3G is a human cytidine deaminase that can potentially hypermutate viral genomes, causing impairment of reverse transcriptase activity during the retrotranscription process (Kaake et al., 2021; Chesarino and Emerman, 2022). Tetherin and A3G expressions might be increased by EVs at the mRNA level of cellular restriction factors (Navarrete-Muñoz et al., 2021).

Current research has demonstrated that EVs can act as the latency reversal agents (LRAs) delivered to target HIV-1 infection cells. Activation of latent viruses and their elimination by cART therapy is currently a hot topic of research for functional cures. Tang et al. (2018) showed that after exogenous Tat protein targeting, the virus reactivated and produced replication-competent HIV-1 in three of the six subjects. Nef and TAR have also been shown to activate the Akt–mTOR and NF-kβ pathways by triggering immunity leading to persistent inflammation (Arenaccio et al., 2014, 2015; Navarrete-Muñoz et al., 2021; Sviridov et al., 2022).

### 4.4. Other treatments

Vitamin D can modulate innate and adaptive immune (Al-Tarrah et al., 2018; Eckard et al., 2018) and maintain intestinal flora balance (Dimitrov and White, 2017). Appropriate exogenous Vitamin D supplementation can promote CD4^+^ T cell proliferation, reduce inflammation and immune activation, prevent PLHIV from developing immune reconstitution inflammatory syndrome, tuberculosis, and mortality, and delay HIV disease progression (Jimenez-Sousa et al., 2018; Currò et al., 2020).

Other studies have demonstrated that probiotics/prebiotics can improve microbial translocation, regulate intestinal microbes, help increase Th17 frequency, and promote CD4 T cell rebuilding (Ortiz et al., 2016; Geng et al., 2020).

## 5. Summary

In conclusion, the process of incomplete immune reconstitution in AIDS is extremely complex and is the result of a multiplicity of factors which can reinforce each other and continuously advance the disease. The most obvious manifestations include a reduction in the number and function of CD4^+^ cells and chronic and sustained immune activation. Different INRs and ISRs may behave in the same way but may have different pathogenic mechanisms, and therefore, individualized treatment for each of these mechanisms is necessary to achieve good outcomes and restore immune reconstitution. Although the mechanisms of incomplete immune reconstitution are currently incompletely elucidated and its application in targeted therapy still faces some difficulties, extensive and ongoing research can help conquer many of these challenges. Therefore, in addition to early cART where possible, clarifying the mechanisms of incomplete immune reconstitution and developing targeted individualized treatment regimens is currently a daunting task in the field of AIDS.

## Author contributions

LR conceived and designed the subject. WZ wrote the article. All authors contributed to the article and approved the submitted version.

## Funding

This work was supported by National Health and Health Commission of Hubei province, China (No. ZY2021M039).

## Conflict of interest

The authors declare that the research was conducted in the absence of any commercial or financial relationships that could be construed as a potential conflict of interest.

## Publisher’s note

All claims expressed in this article are solely those of the authors and do not necessarily represent those of their affiliated organizations, or those of the publisher, the editors and the reviewers. Any product that may be evaluated in this article, or claim that may be made by its manufacturer, is not guaranteed or endorsed by the publisher.
